# Advancements in Melanoma Treatment: A Review of PD-1 Inhibitors, T-VEC, mRNA Vaccines, and Tumor-Infiltrating Lymphocyte Therapy in an Evolving Landscape of Immunotherapy

**DOI:** 10.3390/jcm14041200

**Published:** 2025-02-12

**Authors:** Apoorva Mehta, Mateen Motavaf, Ikenna Nebo, Sophia Luyten, Kofi D. Osei-Opare, Alejandro A. Gru

**Affiliations:** 1 Columbia University Vagelos College of Physicians and Surgeons, 630 W 168th St, New York, NY 10032, USA; idn2106@cumc.columbia.edu (I.N.); stl2148@cumc.columbia.edu (S.L.); kdo2118@cumc.columbia.edu (K.D.O.-O.); 2 Duke University School of Medicine, Durham, NC 27710, USA; mateen.motavaf@duke.edu; 3 Department of Dermatology, Columbia University Irving Medical Center, New York, NY 10032, USA; aag2222@cumc.columbia.edu

**Keywords:** melanoma, treatment, mRNA vaccine, TIL, therapy, T-VEC, PD-1 inhibitors, PD-L1 inhibitors, CAR-T, T-cell, immunotherapy, advancement

## Abstract

Melanoma, an aggressive skin cancer, presents significant therapeutic challenges. Consequently, innovative treatment strategies beyond conventional chemotherapy, radiation, and surgery are actively explored. This review discusses the evolution of immunotherapy in advanced melanoma, highlighting PD-1/PD-L1 inhibitors, mRNA vaccines, Talimogene Laherparepvec (T-VEC), and tumor-infiltrating lymphocyte (TIL) therapies. PD-1/PD-L1 inhibitors such as pembrolizumab and nivolumab block immune checkpoints, promoting T-cell cytotoxic activity and improving overall survival in patients with advanced melanoma. T-VEC, a modified oncolytic herpes virus, promotes a systemic anti-tumor response while simultaneously lysing malignant cells. mRNA vaccines, such as Moderna’s mRNA-4157/V940, take advantage of malignant-cell-specific neoantigens to amplify the adaptive immune response while protecting healthy tissue. TIL therapy is a form of therapy involving ex vivo expansion and reinfusion of the patient’s tumor-specific lymphocytes and has been shown to provide durable tumor control. While these therapies have demonstrated promising clinical outcomes, challenges such as tumor resistance, high financial burden, and limited accessibility pose challenges to their widespread use. This review explores combination therapies such as PD-L1 inhibitors with mRNA vaccines, or TIL therapy, which aim to enhance treatment through synergistic approaches. Further research is required to optimize these combinations, address barriers preventing their use, and control adverse events.

## 1. Introduction

Melanoma, a malignant tumor originating from melanocytes, is one of the most aggressive and treatment-resistant skin cancers. It accounts for the majority of dermatological-related deaths globally [1]. Standard treatments such as surgery, chemotherapy, and radiation therapy have served as first-line treatments for melanoma for decades. However, for patients with advanced or metastatic melanoma, these therapies have exhibited limited efficacy in reducing tumor size, decreasing recurrence, and limiting metastasis [2]. As a result of the rising incidence and public health concern for melanoma, innovative approaches to treatment that may further improve patient prognosis continue to be explored and have shown promising results in clinical trials [3].

Chemotherapies and targeted therapies have long served as the first-line treatment for melanoma. However, some chemotherapeutic agents such as dacarbazine tend to fall short in efficacy or achieve a durable response in patients with advanced disease [4]. Additional shortcomings in melanoma therapies are also observed in radiation therapy; many forms of melanoma rapidly develop radioresistance, rendering radiation-induced cytotoxicity ineffective [5]. As a result of these limitations, researchers began to investigate other forms of treatments, such as immunotherapies. Amongst the first forms of immunotherapy for melanoma were interferon-alpha (IFN) and high doses of interleukin-2 (IL-2). While these therapies demonstrated effectiveness in clinical practice, there were significant systemic adverse effects such as the cytokine toxicity and shock seen in IL-2. Targeted therapies emerged after discovering melanoma-specific tyrosine kinase receptors like BRAF, which have long served as targets for inhibitors such as vemurafenib and dabrafenib. The development of signaling cascade inhibitors has become a popular avenue for melanoma treatment with the creation of MEK inhibitors (trametinib and cobimetinib); however, tumor resistance limits their long-term efficacy [6].

Further immunotherapy advancements were made by introducing checkpoint inhibitors. Cytotoxic T-lymphocyte-associated antigen 4 (CTLA-4) inhibitors such as ipilimumab exert their effects by stimulating early-stage T-cell activation. Although response rates were limited, CTLA-4 inhibitors demonstrated improved overall survival. These discoveries were quickly followed by PD-1 (pembrolizumab and nivolumab) and PD-L1 (atezolizumab) which exert their effects by blocking the PD-1/PD-L1 pathway, allowing T-cells to remain active and amplify their cytotoxic effect [7]. Clinical trials investigating PD-1 and PD-L1 inhibitor efficacy are shown to improve prognosis in patients with metastatic melanoma compared to conventional chemotherapies and radiation therapies. However, tumors often develop tolerance to these inhibitors [7,8]. Another explored form of immunotherapy includes indoleamine 2,3-dixoygenase (IDO) inhibitors (indoximod and epacadostat). They block the degradation of tryptophan to its metabolites by IDO and reduce the immunosuppressive effects on the tumor microenvironment. However, phase III trials such as ECHO-301 exhibited poor survival benefits when combined with adjunctive therapy, raising significant concerns about whether it would even be a viable standalone treatment option [9,10].

Talimogene Laherparepvec (T-VEC) is another more modern therapy route that is effective in melanoma treatment. It was first approved in 2015 and has shown impressive results in treating patients with unresectable, injectable cutaneous, subcutaneous, and nodal lesions. T-VEC leverages a modified form of herpes simplex virus type 1 (HSV-1) to selectively infect and lyse malignant cells. The release of malignant antigens further stimulates an anti-tumor response, amplifying T-VEC’s efficacy. The pivotal OPTiM trial demonstrated that T-VEC improved durable response rates (DRRs) compared to GM-CSF monotherapy for advanced melanoma. A durable response rate (DRR) was seen in 16.3% of patients treated with T-VEC, compared to 2.1% in the control group. However, there were only marginal overall survival improvements, suggesting that T-VEC’s strengths lie in its ability to control localized disease rather than as a systemic solution [11,12].

More recently, Nemvaleukin Alfa, a reformulated IL-2 variant, was developed to retain the anti-tumor benefits of IL-2 while minimizing the cytokine-induced toxicity associated with earlier IL-2 therapies. Early-phase clinical trials have shown promising results, particularly when combined with immune checkpoint inhibitors such as pembrolizumab. However, long-term efficacy and safety are still being investigated through randomized clinical trials [13]. Non-medicinal treatments like Imiquimod cream (Aldara) and limb perfusion therapy have also demonstrated potential in specific cases of melanoma management [14,15]. The results are not as significant as those of other emerging routes of therapy.

As the investigation into melanoma treatment progresses, researchers are exploring the introduction of mRNA vaccines and tumor-infiltrating lymphocyte (TIL) therapies as the next generation of melanoma immunotherapy. Moderna’s mRNA vaccine mRNA-4157/V940 encodes tumor-specific neoantigens that invoke patient-specific immune responses targeting a patient’s unique genetic mutations. Advanced melanoma can be exclusively targeted with mRNA vaccines, sparing damage to healthy tissue [16]. Other forms of personalized treatment, such as TIL, augments a patient’s tumor-infiltrating immune cells and reinfuses them back into the tumor. With the recent FDA approval of Lifileucel, a tumor-infiltrating lymphocyte (TIL) product, TIL therapy continues to be further explored as a potential conjunctive treatment for melanoma [17]. 

This review aims to provide comprehensive insight into the evolution of melanoma treatment, specifically discussing PD-1/PD-L1 inhibitors, T-VEC, mRNA vaccines, and tumor-infiltrating lymphocyte (TIL) therapy. By examining these treatments’ mechanisms of action, efficacy in treatment, impact on prognosis, and potential synergies when used in unison, we hope to highlight the ever-advancing forms of immunotherapy and therapeutic progress in melanoma.

## 2. PD-1/PD-L1 Inhibitors

### 2.1. Mechanisms of Action

Programmed Cell Death Protein 1 (PD-1) inhibitors have introduced a new era of immunotherapy in the management of solid tumors, including melanoma. Landmark trials such as CheckMate-067 and KEYNOTE-006 demonstrated unprecedented response rates in patients with metastatic melanoma, significantly improving overall and progression-free survival [18,19]. These groundbreaking findings led to James Allison receiving the Nobel Prize in Physiology or Medicine for his pioneering work in immune checkpoint inhibition. Inhibitors such as pembrolizumab and nivolumab have significantly improved clinical outcomes and enhanced the body’s tumor-specific immune response by restoring the immune system’s capacity to recognize and eliminate tumor cells. PD-1 inhibitors block the interaction between the PD-1 receptor, expressed on activated T-cells, and its ligands PD-L1/PD-L2, which are commonly overexpressed on tumor cells. This interaction suppresses T-cell activation and allows tumor cells to evade immune detection. By disrupting this pathway, PD-1 inhibitors reinvigorate T-cell activity, enhancing tumor-specific cytotoxic responses. Consequently, cytotoxic T lymphocytes (CTLs) can more effectively recognize, bind to, and lyse malignant cells, resulting in durable anti-tumor responses and improved survival rates in metastatic melanoma [20,21]. PD-L1 inhibitors function by inhibiting programmed death-ligand 1, a transmembrane protein often overexpressed on malignant cells and APCs. Normally, PD-L1 binds to the PD-1 receptor on T-cells, downregulating immunity and promoting immune evasion of tumor cells. By directly preventing their binding, T-cell activation is upregulated, and cytotoxic effector functions take place [22,23,24].

Monoclonal antibodies such as pembrolizumab and nivolumab target the PD-1 receptor on T-cells, inhibiting the receptors’ and ligands’ interactions. Inhibition of PD-1 prevents downstream blockade of ZAP-70 and PI3K/AKT signaling pathways which are critical cascades for T-cell survival effector function [25]. The drug causes T-cells to become more active and leads to greater levels of interferon-gamma (IFN-γ) and tumor necrosis factor-alpha (TNF-a) in the tumor microenvironment. This heightens the immune response against melanoma [26]. Further, PD-1 inhibitors have an additional mechanism involving naïve T-cells. Tumor-associated dendritic cells increase the secretion of chemokines CXCL9 and CXCL10 which recruit naive T-cells [27]. With the increased antigen-presenting cell (APC) activity and increased T-cell count, cancerous antigens are presented to naive T-cells at a higher rate to promote rapid differentiation. Comparatively, unlike PD-1 inhibitors, which focus on the receptor found directly on T-cells, PD-L1 inhibitors target the tumor and antigen-presenting cell, leading to a broader, systemic immune response. 

Expansive growth in cytotoxic T-cell activity leads to increased lysing of melanoma through perforins and granzymes, allowing for durable tumor containment. Ultimately, PD-1 and PD-L1 inhibitors transform the tumor microenvironment from immunosuppressive to one that supports immune engagement and anti-tumor response [28].

### 2.2. Efficacy, Limitations, and Impact on Prognosis

PD-1/PD-L1 inhibitors have come to transform the immunotherapy landscape for solid tumor treatments, especially in melanoma. They enhance the immune response by effectively removing immunosuppressive limitations of the CD8+ effector response. Despite the success of PD-1 therapies in patient prognosis, limitations still exist in efficacy and tumor resistance.

#### 2.2.1. Efficacy in Clinical Trials

PD-1 inhibitors used in cancer immunotherapy have proven successful in various clinical trials, especially in advanced (unresectable or metastatic) melanoma and as an adjuvant therapy for resected stage III melanoma [29,30]. In the phase Ib KEYNOTE-001 trial, patients with unresectable stage III or IV melanoma were randomized to receive pembrolizumab, a PD-1 inhibitor. The 5-year survival (OS) rate was 34% in treatment-naive patients, and 23% in previously treated patients. Further, the median OS for treatment-naive patients was 38.6 months, while for previously treated patients, it was 23.8 months [29]. 

Another clinical trial, KEYNOTE-006 by Long et al., recorded results that align with the findings of the KEYNOTE-001 trial. In stage III or IV melanoma, pembrolizumab, a PD-1 inhibitor, resulted in a median overall survival of 34% compared to ipilimumab monotherapy, which resulted in 23.6% overall survival. These results indicate that patients with melanoma experience a marked survival benefit when PD-1 inhibitors are used [30].

Neoadjuvant (pre-surgical) use of PD-1 inhibitors has also exhibited significant results in clinical trials. A pooled analysis of six clinical trials involving 573 patients with high-risk resectable melanoma compared the safety and efficacy of neoadjuvant combination therapy with anti-PD-1 and anti-CTLA-4 inhibitors to anti-PD-1 monotherapy. The results indicated that while dual checkpoint inhibition was associated with an increased pathological response, anti-PD-1 monotherapy provided a more favorable safety profile [31]. Continued neoadjuvant trials are ongoing at a large scale, which is evident through a global analysis of neoadjuvant and adjuvant anti-PD-1/PD-L1 clinical trials demonstrating a high prevalence in melanoma-specific research [32]. Successful further investigation into neoadjuvant PD-1/PD-L1 inhibitor therapy can improve post-surgical outcomes and promote overall survival in patients with advanced melanoma.

PD-L1 inhibitors have also demonstrated efficacy in clinical trials, particularly when used in combination therapies. Atezolizumab, a PD-L1 inhibitor, received FDA approval in 2020 to be used with cobimetinib (MEK inhibitor) and vemurafenib (BRAF inhibitor) in patients diagnosed with BRAF V600 unresectable or metastatic melanoma. This combination therapy is indicated in patients with BRAF V600 mutated melanoma. Further, this combination therapy is contraindicated in patients with BRAF wild-type melanoma, as there have been no recorded benefits. The IMspire150 phase III trial evaluated the safety and efficacy of atezolizumab in combination with vemurafenib and cobimetinib and found a median progression-free survival (PFS) of 15.1 months compared to 10.6 months with vemurafenib and cobimetinib alone [33]. A monotherapy trial assessing atezolizumab in metastatic melanoma reported a median overall survival (OS) of 36% with a disease control rate of 45%, ultimately determining that patients with higher PD-L1 expression correlated with better therapy response [34]. 

#### 2.2.2. Efficacy in Cold Tumors

Cold tumors are described as tumors with low immunogenicity with minimal T-cell infiltration [35]. The efficacy of PD-1 inhibitors in cold tumors is often significantly lower: a critical component of PD-1 inhibitor efficacy is the availability of a large pool of activated CD8+ cytotoxic T-cells. As a consequence, PD-1 inhibitors are not indicated for tumors with low TMB or “cold” profiles due to poor efficacy when modulating immune checkpoint inhibitors [28]. With low T-cell infiltration, the therapeutic potential of PD-1 inhibitors cannot be realized. Additionally, a high concentration of regulatory T-cells and myeloid-derived suppressor cells exist in the tumor microenvironment, further attenuating the immune response [36]. Beyond the limited availability of CD8+ cells, a dense tumor stroma further prevents infiltration, limiting an effective immune response. Fibroblasts in the tumor microenvironment also secrete cytokines such as TGF-B which are downstream regulators of CD8+ effector functions [35,36]. As a result of these limitations, research published by Habibi et al. underscores the necessity for combination therapies to overcome the immunosuppressive nature of cold tumors [37].

Investigators have conducted trials to determine whether IDO inhibitors such as epacadostat would be effective when used in conjunction with PD-1 inhibitors since IDO itself is an immunosuppressive enzyme. Although promising on paper, the ECHO-301/KEYNOTE-252 trial involving patients with metastatic melanoma found no significant improvements in progression-free survival or overall survival compared to standalone PD-1 inhibitor therapies [10]. While modulating the IDO enzyme’s activity remains an interesting avenue of treatment, further investigation is required. 

PD-L1 inhibitors such as atezolizumab and durvalumab have also shown low efficacy in cold tumors. The low immunogenicity of such tumors, coupled with a lack of pre-existing immune activity, limits the effectiveness of PD-L1 inhibitors. Consequently, ongoing research has pivoted to using PD-L1 inhibitors in combination therapies that aim to convert the tumor environment into one that is “hot”. A study published in *Frontiers in Immunology* discusses using PD-L1 inhibitors with cancer vaccines to convert tumors into more immunogenic forms, though data are still limited [38]. While PD-L1 inhibitors have demonstrated remarkable efficacy in advanced melanoma patients with “hot” tumor microenvironments, their effectiveness in cold tumors requires further investigation [39]. 

#### 2.2.3. Tumor Mutational Burden (TMB) and Efficacy

Patients with low-mutational-burden tumors present a distinct therapeutic challenge, as these tumors generate insufficient neoantigens with genetic variety to elicit robust T-cell responses. In contrast, melanoma is generally considered to have high TMB, predisposing the cells to more favorable treatment [40]. However, given the wide variety of melanoma phenotypes, not every subtype exhibits a favorable high mutational burden. For example, triple wild-type melanomas, which are melanomas lacking mutations in BRAF, NRAS, and NF1, exhibit markedly low TMB compared to NF1-mutant melanomas. This directly relates to the difficulty in the characterization and treatment response of triple wild-type melanomas. Similarly, subtypes such as acral and mucosal melanomas also tend to have lower TMBs, which tend to cause providers to contraindicate PD-1 monotherapy. This variability in TMB underscores a clinician’s challenge in assessing the patient’s likelihood of benefitting from PD-1 therapy and considering a patient’s unique disease state and mutations in assessing potential treatment response [40].

#### 2.2.4. Tumor Resistance Against PD-1 Inhibitors

Two forms of tumor resistance can develop: primary and acquired. Primary resistance describes the inherent lack of ability to respond to PD-1 and PD-L1 inhibitors due to the tumor microenvironment, primarily through insufficient PD-1/PD-L1 expression, disruption of critical immune signaling pathways, or lower tumor mutational burden. Acquired resistance pertains to resistance that develops due to adaptive mutations made by the tumor cells [41]. Such adaptive mutations can include dismantling MHC Class I molecules through the deletion of beta-2 microglobulin (B2M), an essential component of the MHC complex. Without B2M, tumor cells develop immunoevasive ability, making PD-1/PD-L1 inhibitors futile. Tumor cells may also develop the ability to upregulate other checkpoints, such as TIM-3, LAG-3, or VISTA, which attenuate the immune response further [41,42]. Among patients who have melanoma, which is known to have one of the highest rates of response to immune checkpoint inhibitors, 60–70% of patients still fail to achieve a measurable response to treatment, and of those that do achieve a response, 20–30% relapse and experience tumor progression [43].

#### 2.2.5. Immune-Related Adverse Events

PD-1/PD-L1 inhibitors inadvertently put patients at risk of immune-related adverse events (irAEs) due to the overactivation of the immune system. They have been linked to the onset or exacerbation of type 1 diabetes mellitus (T1DM), which is characterized by the T-cell mediated destruction of pancreatic beta cells [44,45]. This occurs because the PD-1/PD-L1 blockade prevents the interaction between PD-1 on autoreactive T-cells and PD-L1 on pancreatic beta cells, an interaction that usually stymies immune-mediated damage. A case review analysis found that among 2960 patients treated with immune checkpoint inhibitors, 27 cases (0.9%) developed insulin-deficient diabetes; 59% of these 27 patients also experienced diabetic ketoacidosis, and 85% experienced rapid loss of pancreatic beta cell function, which is evident through the rapid development of a hyperglycemic state [46]. Importantly, this form of diabetes occurs more frequently with anti-PD-1 and anti-PD-L1 therapies than with other forms of immune checkpoint inhibitors, underscoring the specific risks associated with PD-1 /PD-L1 inhibition. Similarly, systemic lupus erythematosus (SLE), multiple sclerosis (MS), interstitial lung disease (ILD), and inflammatory bowel disease (IBD) have been exacerbated in patients treated with PD-1/PD-L1 inhibitors [47,48,49,50]. Dermatological manifestations of PD-1/PD-L1 blockade occur in 30–60% of patients. These cutaneous adverse events include dermatitis, lichenoid reactions, and psoriasis. Consequently, these therapies are commonly contraindicated in patients with autoimmune diseases such as systemic lupus erythematosus (SLE), multiple sclerosis (MS), and inflammatory bowel disease (IBD). These adverse effects significantly impact the quality of life of patients and often lead to the discontinuation of therapy [51,52,53]. These risks demand meticulous patient selection, biomarker monitoring, and symptom management when providers decide to use PD-1/PD-L1 inhibitors in melanoma treatment.

In summary, while PD-1/PD-L1 inhibitors have shown benefits in clinical practice, there are significant barriers to efficacy when used alone. Combination therapy should be considered for checkpoint inhibitors to promote a more significant therapeutic effect.

## 3. T-VEC Immunotherapy

Talimogene Laherparepvec (T-VEC) uses a modified oncolytic herpes virus to treat unresectable advanced and metastatic melanoma via intralesional injections. Commercialized by Amgen Inc. under the name of Imlygic, T-VEC is the first oncolytic virus and intralesional therapy to have received approval from both the U.S. Food and Drug Administration (FDA) and European Medicines Agency (EMA) [54].

### 3.1. Mechanisms of Action

#### Modifying Herpes Simplex Virus Type 1 (HSV-1)

T-VEC’s ability to promote anti-tumor immunity is what makes it a first-in-class immunotherapy. Genetically engineered herpes simplex virus type 1 (HSV-1) selectively targets tumor tissue to lyse tumor cells while also enhancing anti-tumor immunity [55,56]. Fortunately, HSV has been genetically modified for cell type selectivity. Functional deletions in neurovirulence factor ICP34.5 reduce viral pathogenicity which allows the virus to selectively replicate in tumors. ICP47 attenuates viral-mediated suppression of antigen presentation and promotes virus growth in cells, by increasing the expression of HSV US11, without impacting tumor selectivity. Finally, a human granulocyte–macrophage colony-stimulating factor (GM-CSF) gene is inserted into the HSV-1 for local production [57].

T-VEC uses genetically modified HSV-1 to target cancer cells using glycoproteins gB and gC and triggers cell lysis [58,59]. The GM-CSF from genetically modified HSV-1, viral particles, and expelled tumor-derived antigens are released via lysis which facilitates the infection of surrounding tumor cells and enhances the spread of tumor cell antigens [59]. The primed CD8+ T-cells that are then recruited to the intra-tumoral site of injection will produce a system polyclonal anti-tumor response [60,61].

No pharmacologic studies have been performed since T-VEC is administered via intra-tumoral injections. However, it is important to factor in that mouse models have shown that high concentrations of viral DNA are detectable around the site of injection. Viral DNA is found in low concentrations in peripheral sites such as the heart, liver, lymph node, kidney, spleen, and blood while it was otherwise undetected in the nasal mucosa, feces, bone marrow, and eyes [55]. Lastly, the genetically modified HSV-1 is normally eliminated via normal host–defense mechanisms, which include immune responses and autophagy, but viral DNA occasionally persists in neuronal cell bodies. As of today, this does not seem to present any clinical implications, but more research is required [55].

### 3.2. Efficacy, Limitations, and Impact on Prognosis

#### 3.2.1. Efficacy of Monotherapies

The use of T-VEC as a monotherapy has yielded promising results. T-VEC was first tested in a phase I study where it was able to demonstrate GM-CSF expression, tumor apoptosis and necrosis, inflammation and erythema, and viral replication upon analysis of post-treatment biopsies in patients with unresectable stage IIIB, IIIC, or IV melanoma with injectable cutaneous, subcutaneous, or nodal lesions. The therapy had maintained a low grade and acceptable toxicity profile with fever, chills, local reactions, and myalgia being the only adverse effects reported [55,62].

A phase II study was conducted on 50 patients with locally advanced or metastatic melanoma. There was an overall response rate (ORR) of 26%, which included five partial responses (PRs) and eight complete responses (CRs). In total, 92% of these responses were maintained for around 3 years. Overall survival (OS) was reported to be 58% at 12 months and 52% at 24 months [63].

Another study conducted by Kaufman et al. showed that T-VEC could induce robust local and systemic CD8+-mediated immunity in melanoma patients with advanced disease [64]. Tumor-infiltrating lymphocytes produced more IFN-gamma and MART-1 CD8+ T-cells, along with inducing a decrease in FoxP3+ T-cells, in the injected melanoma lesions post-injection when compared to the untreated melanoma control. The group also demonstrated that myeloid-derived stem cells significantly decreased in number in injected lesions when compared with non-injected lesions in the same patient or in a control patient [64].

A phase III OPTIM study randomized 436 patients with locally advanced or metastatic melanoma to either receive intralesional T-VEC or subcutaneous GM-CSF [12]. Researchers reported significantly higher DRR with the T-VEC treatment (19.3) compared with GM-CSF (1.4). Notably, 16.9% of T-VEC-treated patients achieved complete response (CR) compared to only 0.7% for GM-CSF-treated patients. In addition, 14.6% of T-VEC-treated patients achieved partial response (PR) compared to only 5.7% of GM-CSF-treated patients. T-VEC-treated patients reduced the risk of death by 21% when compared to GM-CSF-treated patients. They also determined that the T-VEC toxicity profile was acceptably low since the most common adverse events were fatigue, chills, and pyrexia (flu-like symptoms) [12].

A multi-institutional review study in 2022 highlighted the potential for T-VEC to be used as a salvage pathway for patients with narrowed treatment options after ineffective immunotherapy. The study demonstrated that treating unresectable melanoma was effective as they achieved 14% PR and 37% CR. They determined that the median overall disease-free survival (DFS) after CR was 32 months, and the median-in-field progression-free survival (PFS) was 15 months. Ultimately, these findings continue to prove the effectiveness of T-VEC as a means of managing unresectable, metastatic melanoma, especially in patients who are not candidates for surgery or other systemic therapies [65].

#### 3.2.2. Limitations

Since T-VEC is a novel oncolytic immunotherapy, there are significant treatment-associated costs that limit patient access [66]. The handling, storage, and transportation of T-VEC is particularly burdensome. T-VEC has to be stored in a solid state at −90 to −70 °C and has to then be thawed to a liquid state before preparation which can take between 30 and 70 min. Pharmacy workflow must be modified so technicians have the time to prepare the syringes and IV hoods. Some major barriers to implementation are a shortage of providers who are trained to administer T-VEC, the availability and capacity of freezers, and scheduling [67].

There is also always a fear that the virus can rapidly mutate to become pathogenic. Trials have not shown any HSV-1 acquiring pathogenicity. Current and past trials have shown limited adverse effects outside of flu-like systems when used as a monotherapy. Additional symptoms in combinatorial therapies seem to rather be attributed to the adverse effect profile of the other therapies. Despite this, T-VEC should be used sparingly in patients with autoimmune diseases or in those on high-dose immunosuppressants, as viral-based therapies pose substantial risks in these populations [68].

In summary, T-VEC represents a significant innovation in cancer immunotherapy as the first FDA- and EMA-approved oncolytic virus therapy. However, there are still significant logistical and production challenges to allow for widespread patient access and treatment.

## 4. mRNA Vaccines

### 4.1. Mechanisms of Action

The recent development and application of mRNA vaccines in immunotherapy has pushed the frontier of melanoma treatment. The mechanism of action involves encoding tumor-specific neoantigens, promoting cellular uptake, neoantigen presentation, and amplifying the patient’s adaptive immunity.

Somatic mutations unique to a patient’s melanoma have allowed investigators to develop mRNA vaccines encoding tumor-specific neoantigens. Exposing a patient’s immune system to these neoantigens leads to precision targeting of malignant tumor cells. Healthy tissue therefore avoids damage during treatment and enforces a tumor-specific immune response [69].

Lipid nanoparticles (LNPs) are used to deliver treatment by encapsulating the mRNA and protecting it from protease degradation. With encapsulation, LNPs can safely deliver the mRNA to APCs via endocytosis.

After endocytosis, the mRNA enters the cytoplasm. Host cell ribosomes assimilate onto the mRNA and translate the encoded tumor-specific neoantigens. The neoantigen–MHC complexes then interact with cytotoxic CD8+ T-cells and CD4+ helper T-cells, kickstarting the effector portion of the immune response [70,71]. The TCR–MHC–antigen complex evokes the immune system to form a specific immune response against melanoma cells that contain the same neoantigens [71].

After neoantigen recognition, cytotoxic T lymphocytes proliferate from activating CD8+ T-cells. These lymphocytes specifically target and induce apoptosis in melanoma cells that have the same neoantigen by which the lymphocyte was primed. Tangentially, cytokine and chemoattractant release by CD4+ helper T-cells amplifies the cytotoxic immune response of CD8+ T-cells and recruits other immune cells. This process occurs entirely while sparing healthy tissue, an advantage other forms of therapy do not possess [72]. Beyond the immediate immune response, mRNA vaccines develop immunological memory to prime the immune system against future tumor cells. This characteristic of mRNA vaccines is pivotal as it protects the patient through an avenue once left undefended by conventional therapies [73]. 

mRNA vaccines ultimately exert their effects through encoding tumor-specific neoantigens, endocytosis by antigen-presenting cells, and the activation of the patient’s immune system. By leveraging both an acute and memory response, mRNA vaccines can defeat melanoma tumor cells more comprehensively than traditional chemotherapy and radiation therapy, as highlighted by the Phase2b KEYNOTE-942 trial on mRNA-4157/V940, combined with pembrolizumab [74].

### 4.2. Efficacy, Limitations, and Impact on Prognosis

Personalized mRNA vaccines used in melanoma therapy have proven promising in clinical investigations. Yet, physicians and patients still face challenges regarding efficacy, accessibility, and scalability. 

#### 4.2.1. Efficacy in Clinical Contexts

There are multiple ongoing clinical trials researching mRNA vaccines as a melanoma therapy primarily in patients with advanced or metastatic melanoma. BNT111 is a leading candidate targeting melanoma tumor-associated antigens in a current phase II trial which previously showed induction of both CD4+ and CD8+ T-cell responses in 78% of patients, both alone and in combination with cemiplimab, a PD-1 inhibitor [75]. Two personalized mRNA cancer vaccines, mRNA-4157 and BNT122, are also in phase II clinical trials. The BNT122 vaccine demonstrated induction of neoantigen-specific T-cell responses in 50% of patients with pancreatic ductal adenocarcinoma in the phase I trial [76]. mRNA-4157 demonstrated toleration and similar induction of neoantigen-specific T-cell responses, but the data have not yet been published in a peer-reviewed journal [77]. There is significant potential in these developing mRNA vaccines: if clinical trials prove further efficacy, mRNA vaccines would be highly indicated for patients with resectable or unresectable melanoma, specifically in high-risk patients or in patients with treatment-refractory disease.

#### 4.2.2. Vaccine Development, Scaling, and Financial Burden

Despite their efficacy, developing personalized mRNA vaccines is costly and time-intensive and the involved R&D poses significant barriers to scaling and clinical application [78]. As a first step, neoantigens from patients’ tumors need to be extracted, sequenced, and identified [69,70,71]. It is important to note that during vaccine preparation, only a small fraction of the tumor is typically sampled. In melanoma, a cancer known for rapid growth, high mutation rate, and heterogeneity, this small sample is unlikely to capture the full diversity of the cancer [79]. If the encoded antigens are then only expressed in a subset of the tumor cells, resistant or untargeted tumor cells can evade immune detection. This emphasizes a relative contraindication of using mRNA vaccines against tumors that exhibit low neoantigen burden. Another issue is the selection of neoantigens that will generate robust and sustainable immune responses. While mRNA vaccine preparation itself may be quicker than alternatives, identifying the most clinically relevant neoantigens can significantly extend vaccine preparation timelines. This is further complicated by human leukocyte antigen (HLA)-type heterogeneity amongst the population that dictates antigen binding and immune response to the vaccine, thus generally leading to contraindication in patients with rare HLA subtypes that limit antigen binding and T-cell activation [80]. Lastly, LNPs, the primary delivery system for mRNA, are difficult to manufacture at scale. A precise balance of lipids, encapsulating agents, and RNA is necessary within a controlled environment to produce stable particles of the correct size and structure. This is problematic at scale given that minor deviations in the formulations can lead to unstable particles or diminished therapeutic efficacy [81].

mRNA vaccine development requires specialized personnel, costly equipment, and significant time investment. As a result, national access to these treatments is severely limited [82,83]. In patients with advanced or metastatic melanoma, these logistical roadblocks underscore the predicament of pursuing these therapies as a first-line treatment [84]. Combined with regulatory requirements imposed by the FDA, the prompt application of mRNA vaccines in clinical practice is increasingly challenging without new regulations to accelerate development and assure safety [82,83].

Ultimately, while mRNA vaccines have shown promising results in clinical trials, these challenges must be addressed for them to tangibly impact melanoma prognosis.

## 5. Tumor-Infiltrating Lymphocyte (TIL) Therapy

### 5.1. Mechanisms of Action

Tumor-infiltrating lymphocyte (TIL) therapy involves an adoptive cell transfer (ACT) of the patient’s own T-cells into their tumor. The main objective of this therapy is to artificially increase the number of tumor-specific T-cells to control tumor cell proliferation [85].

These T-cells are already differentiated and specialized to target tumor antigens [86]. Although these T-cells have an elevated density, they are often ineffective due to the high levels of immune checkpoint receptor activity, such as PD-1 and CTLA-4 [87]. Investigators have conducted trials examining the clinical impact of the CTLA-4 blockade. The use of agents such as ipilimumab, a CTLA-4 inhibitor, amplified the efficacy of TIL therapy by preventing T-cell suppression but had significantly higher immune-related adverse events [86,87].

In ex vivo, a culture is created that favors the proliferation of T-cells specific to melanoma antigens. Interleukin-2 (IL-2) is also added to the culture, which aids in the proliferation of tumor-specific T-cells. Critical cytotoxic molecules such as perforin and granzymes are also upregulated [86].

Patients undergo lymphodepletion prior to the reinfusion of an expanded collection of TILs. Lymphodepletion removes existing regulatory T-cells (Tregs) and myeloid-derived suppressor cells (MDSCs) to accommodate the infused TILs. Lymphodepletion also upregulates the release of IL-7 and IL-15, which promotes TIL survival and effector function [86].

TILs are then infused into the patient. Because of ex vivo exposure to melanoma antigens, infused TILs localize rapidly to the tumor microenvironment to exert their effector function. The adaptive immune response is also stimulated by differentiating TILs into memory T-cells. This allows for an amplified acute response and sustained prevention of tumor neogenesis [88].

TIL therapy highlights how a patient’s existing immune response can be enhanced through the expansion of T-cells directly harvested from the patient’s tumor and effectively personalize their treatment.

### 5.2. Efficacy, Limitations, and Impact on Prognosis

TIL therapy has shown significant benefits in prognosis in patients suffering from advanced and metastatic melanoma. However, challenges exist both at the patient and macro level.

#### 5.2.1. Efficacy

TIL therapy clinical trials have shown promising data and are especially indicated for patients who have experienced disease progression [89,90]. The phase II C-144-01 trial of Lifileucel, an autologous TIL therapy, reported an objective response rate (ORR) of 31.4% amongst a patient population of 153. At a median follow-up of 27.6 months, the median duration of response (DOR) was not reached, emphasizing the ability to mount a durable immune reaction. Additionally, at the 18-month mark, 41.7% of responses were maintained. These results show promise for the potential of TIL to improve clinical outcomes while maintaining a safe risk profile. As a result of the decreased risk profile, TIL therapy is currently being investigated as a primary treatment for patients with refractory disease who have limited systemic therapy options [90].

#### 5.2.2. Challenges in Application and Scalability

The steps involved in TIL therapy are intensive and require extensive patient preparation. The patient must first undergo surgical resection to obtain tumor tissue to isolate tumor-specific lymphocytes, which are then amplified in vitro. Advanced tumors, such as those in visceral or inaccessible sites, may not yield sufficient tissue for isolation. Patients with low tumor burden or non-resectable visceral metastases are often unsuitable candidates to receive this treatment [91]. Patients then undergo lymphodepletion before TIL infusion to improve the engraftment of infused TILs; this requires patients to be healthy enough to tolerate both the surgical procedure and immune system attenuation. TIL therapy is commonly contraindicated in patients with severe organ diseases, infections, or autoimmune diseases due to the impact of lymphodepletion and immune reconstitution. Patients suffering from comorbidities or with significant systemic symptoms from advanced and/or metastatic melanoma are therefore often unsuitable for the treatment due to the increased risk profile [92,93,94].

The scalability of TIL therapy is also highly complex, costly, and labor-intensive: in vitro expansion takes weeks to months to achieve a lymphocyte count that can mount a meaningful immune response. Once the expanded pool of TILs has been generated, TIL therapies undergo several quality assessment protocols to reduce patients’ adverse effects [95]. These protocols include sterility testing (confirming no microbial species contamination), viability assays (ensuring TILs perform their effect function), and potency tests (evaluating cytotoxicity and efficacy of effector function). Further evaluations, such as flow cytometry and T-cell receptor analyses, confirm the presence of T lymphocytes that target neoantigens of the tumor specifically [92,96]. This highly specific and labor-intensive manufacturing drives up costs and makes it inaccessible to many patient populations and health systems [95].

While TIL therapy’s personalized approach towards melanoma therapy improves patient prognosis and provides a durable tumor response, widespread adoption remains difficult. Unlike off-the-shelf therapies like checkpoint inhibitors, the highly patient-specific nature of TIL therapy makes it difficult for large-scale utilization without increased cost optimization and technological advancement [94]. Addressing the challenges of scalability and development will be critical for future use cases.

## 6. Synergies Between Therapies

Combining immunotherapies has been explored as a potential strategy to improve treatment outcomes in melanoma, particularly for patients with high-risk or advanced disease. By leveraging different approaches including the mRNA-4175/V940 therapy or tumor-infiltrating lymphocyte (TIL) therapy in combination with anti-PD-1 checkpoint inhibitors like pembrolizumab or nivolumab, anti-tumor immune responses can be enhanced. However, the success of such approaches varies, and not all combinations have demonstrated clear clinical benefits.

### 6.1. MEK Inhibitors + PD-1/PD-L1 Inhibitors

The use of MEK inhibitor and PD-1 inhibitor combination therapy has been investigated thoroughly in patients with advanced melanoma, specifically among patients with BRAF V600 mutations.

A phase I trial by Chénard-Poirier et al. evaluated the use of MEK inhibitor selumetinib and PD-1 inhibitor pembrolizumab in patients with advanced melanoma. The results indicated that the combination therapy’s risk profile was relatively well tolerated, with minimal immune-related adverse effects. Common adverse events included rash (53.1% of patients affected), diarrhea (46.9%), and fatigue (43.8%). Although this study focused on risk profile and safety, the study reported an objective response rate (ORR) of 25% [97].

Another study in *Frontiers* evaluated the efficacy of triplet combination therapy with BRAF inhibitors, MEK inhibitors, and PD-1/PD-L1 inhibitors in patients with metastatic melanoma. The meta-analysis discusses five randomized controlled trials containing 1266 patients with stage III-IV melanoma. The study reports that triple combination therapy resulted in a decreased mortality rate compared to existing combination therapies for melanoma and monotherapy. However, more immuno-modulation resulted in a higher incidence of side effects, highlighting the bottleneck that comes with this form of therapy [98]. 

Alternatively, some studies demonstrate that doublet or triplet combination therapies involving PD-1/PD-L1 inhibitors, MEK inhibitors, or BRAF inhibitors have limited clinical efficacy compared to targeted monotherapies. A study published in the *European Journal of Cancer* reported that triplet therapy did not have any substantial advantage in patients with metastatic melanoma compared to targeted treatment [99].

While MEK inhibitor and PD-1 inhibitor combination therapy presents a promising avenue to pursue, clinical trial outcomes have remained controversial. Additional research must be conducted to identify patient populations in which such treatments would be beneficial.

### 6.2. CTLA-4 Inhibitors + PD-1/PD-L1 Inhibitors

PD-1 and CTLA-4 inhibitors in combination therapy have shown significant clinical efficacy in treating advanced melanoma compared to monotherapies. T-cell activation is augmented by preventing immune suppression through PD-1 and CTLA-4 blockades.

A phase III study, the CheckMate-067 trial, investigated the efficacy of the PD-1 inhibitor nivolumab with the CTLA-4 inhibitor ipilimumab compared to monotherapies in patients with advanced melanoma who had not received treatment. The results indicated a 3-year overall survival (OS) rate of 58% compared to 52% in nivolumab alone and 34% in ipilimumab alone. Median progression-free survival (PFS) was 11.5 months for the combination therapy, nearly double that of nivolumab alone at 6.9 months and 2.9 months for ipilimumab alone. Moreover, the combination therapy’s objective response rate (ORR) was 61%, a significant increase from 44% with nivolumab monotherapy [100]. Another study, the CheckMate-069 trial, also assessed the same combination therapy in patients with advanced melanoma and reported an ORR of 61% compared to 11% in ipilimumab monotherapy. However, adverse events were significant, with 59% of patients suffering from grade 3 or 4 treatment-related adverse events. The most common adverse events were colitis (11% of patients), hypophysitis (2% of patients), and diarrhea (4% of patients) [101].

More recently, a 10-year outcome study discussing nivolumab + ipilimumab combination therapy provides more substantial data. The median overall survival (OS) was 71.9 months with the combination therapy compared to 36.9 months for nivolumab alone and 19.9 months for ipilimumab alone. With 37% of patients alive at the end of the trial, melanoma-specific survival exceeded 120 months [102]. 

While the PD-1 and CTLA-4 inhibitor combination therapy provides clinically significant survival benefits and outcomes, a relatively high-risk profile remains. Investigation is still required into which patient populations benefit the most from this combination therapy, emphasizing the importance of personalized treatment in melanoma. 

### 6.3. mRNA Vaccine + PD-1 Inhibitors

The combination of mRNA vaccines and PD-1 inhibitors has shown promise in enhancing melanoma treatment outcomes. The KEYNOTE-942 trial investigated the efficacy of pembrolizumab, an anti-PD-1 therapy, in combination with the individualized neoantigen mRNA-4157 (V940) vaccine [74]. This phase IIb open-label, randomized trial compared the efficacy of combination therapy to pembrolizumab monotherapy in patients with completely resected high-risk cutaneous melanoma. The results demonstrated that recurrence-free survival and metastasis-free survival were longer in the combination therapy group as compared to pembrolizumab monotherapy, with a recurrence hazard ratio of 0.561 (95% CI 0.309–1.017). Moreover, the combination group experienced a lower recurrence and death rate (22% vs. 40%) and a higher 18-month recurrence-free survival of 79% (95% CI 69.0–85.6) compared to 62% (95% CI 46.9–74.3). Importantly, the safety profile was comparable, with adverse events reported in 36% of patients in both groups. These researchers hypothesized that the mRNA-4157 neoantigen therapy enhanced the anti-PD-1 checkpoint therapy by increasing both endogenous and de novo T-cell responses [74].

The KEYNOTE-603 phase I study assessed the same mRNA-4157/V940 individualized neoantigen therapy in combination with anti-PD-1 pembrolizumab or as monotherapy in patients with completely resected stage II–IV cutaneous melanoma [103]. This study corroborated the benefits and results observed in the KEYNOTE-942 trial, with similar clinically meaningful benefits and safety profiles. In addition, the study evaluated immunogenicity, showing that neoantigen-specific T-cell responses elicited the release of IFN-γ and TNF-α. Interestingly, patients treated with pembrolizumab alone exhibited low levels of neoantigen-specific T-cell responses both before and after treatment. The introduction of the mRNA vaccine appeared to expand new T-cell clones that were undetectable following anti-PD-1 therapy alone [103].

### 6.4. TIL Therapy + PD-1 Inhibitors

The combination of TIL therapy with anti-PD-1 inhibitors is another promising avenue for melanoma treatment. In a clinical trial involving 11 patients with stage III/IV metastatic melanoma, an ECOG status of 0–1, and no previous treatment, researchers evaluated the efficacy of nivolumab, an anti-PD-1 therapy, in combination with adoptive cell transfer (ACT) using TILs expanded with anti-4-1BB agonism. The ORR was 36% and the median progression-free survival was 5 months. Notably, two of the patients were non-responders who developed new metastatic lesions, with suspected mechanisms of therapeutic resistance being clonal divergence and intrinsic TIL dysfunction. The paper concluded that the combination therapy of ACT with TIL using anti-4-1BB with anti-PD-1 therapy was a safe and feasible treatment, offering a potential treatment for metastatic melanoma [104].

Similarly, a phase I/II study conducted at Nantes University Hospital investigated a regimen involving initial anti-PD-1 therapy, two subsequent injections of TILs, and a second round of anti-PD-1 therapy. Among the four patients receiving this combination, three achieved objective responses, with complete responses observed in three of these cases [105].

Despite these promising outcomes, prior exposure to anti-PD-1 therapies may reduce the effectiveness of TIL therapy. A retrospective study by Seitter et al. explored the impact of prior anti-PD-1 or MAPK inhibition on ACT-TIL outcomes in patients with metastatic melanoma. They found that patients previously treated with anti-PD-1 or anti-MAPK therapy had a significantly less durable response to ACT-TIL. Proposed mechanisms included reduced mutational burden, altered neoantigen presentation, and upregulation of co-inhibitory receptors. They also hypothesized that the patients enrolled in this trial who were resistant to aPD-1 therapy had immuno-resistant tumors that did not have tumor-specific TILs. Ultimately, they concluded that waiting to use ACT-TIL until after anti-PD-1 treatment may decrease the likelihood of response [106].

This observation was further supported by a study comparing TIL therapy outcomes in anti-PD-1-naive and anti-PD-1-experienced patients. Among 112 naive patients and 69 with prior anti-PD-1 exposure, naive patients exhibited a higher prevalence of neoantigen-reactive T-cells, suggesting that prior anti-PD-1 therapy might impair the immunological landscape necessary for TIL efficacy [107].

Although the combination of TIL therapy and mRNA vaccines represents an intriguing area of research, no clinical trials have yet investigated their synergistic potential. Future studies exploring this combination may provide insights into novel immunotherapeutic strategies for melanoma.

### 6.5. TIL Therapy + mRNA Vaccines

There are currently no publicly reported clinical trials that have tested the combination of tumor-infiltrating lymphocytes (TILs) and the individualized neoantigen mRNA-4157 (V940) therapy for the treatment of melanoma. A combination of these therapies could potentially amplify the immune response of these patients, but this requires further research (Table 1).

## 7. Future Directions

The convergence of PD-1 inhibitors, T-VEC, mRNA vaccines, and TIL therapy into a cohesive treatment plan has the potential to address the limitations of each therapy individually given their complementary mechanisms of action. The mRNA vaccine, with its ability to leverage tumor-specific antigens in the production of neoantigen-specific T-cells, would equip the immune system with T-cells more capable of recognizing and attacking melanoma cells. Given melanoma’s generally high mutational burden, the mutations provide a plethora of potential targets for the vaccine. T-VEC can similarly promote anti-tumor immunity through intralesional injection of genetically modified HSV-1 to recognize and attack melanoma cells while preserving healthy tissue. In tandem, TIL therapy could potentially be used to amplify tumor-specific T-cells, which, already primed to recognize melanoma cells, would be able to mount a more robust immune response. Finally, PD-1 inhibitors would augment the activity of these T-cells through the prevention of T-cell exhaustion even in immunosuppressive tumor microenvironments. 

Key to this integration will be the use of predictive biomarkers to ensure the effectiveness of these specialized therapies. Biomarkers such as the degree of lymphocytic infiltration, autoantibodies, and PD-L1 expression can help clinicians tailor therapies to the specific characteristics of each tumor. Especially within the context of combination therapy, the presence or absence of biomarkers may inform a clinician on how effective the regimen will be—for example, minimal PD-L1 pointing to potential complications with PD-1 inhibition therapy. Future clinical trials will also need to explore the optimal timing, sequencing, and combination dosage of these therapies. Should mRNA vaccines be administered before or after TIL therapy? Should PD-1 inhibitors be used concurrently with these approaches or as maintenance therapy following tumor reduction? Trials will need to evaluate not only the efficacy of combination regimens but also their safety and tolerability, as each of these therapies has associated adverse effects and toxicities. Assessing the durability of response is also of utmost importance in designing future projects, given the tendency of melanoma to relapse.

Cost barriers need to be considered to ensure the feasibility of these therapies. The average cost of the one-time TIL therapy for melanoma, Lifileucel, is USD 515,000, according to the manufacturer. The list price for each indicated dose of PD-1 inhibitor pembrolizumab, when given every 6 weeks, is USD 23,138.32. While the cost for the patient of mRNA cancer vaccines for melanoma is still unknown to the public, it stands to reason that these personalized therapies will also be costly. It is worth noting that there is reason to believe that given their efficacy, these therapies will likely be cost-effective compared to the status quo [108,109,110]. However, making these therapies much more affordable will require innovative solutions such as developing scalable manufacturing processes, patient-friendly healthcare policies, and pathways to integrate these approaches into existing healthcare systems more efficiently. 

Beyond the already discussed novel therapies, Chimeric Antigen Receptor (CAR) T-cell therapy is a newer treatment modality demonstrating early promise for patients unresponsive to previous forms of melanoma immunotherapy. CAR T-cell therapy genetically modifies patient T-cell receptors to precisely target antigens associated with malignant cells, subsequently enhancing the immune system’s ability to recognize and attack cancer cells [111,112,113]. Recent studies are focused on how to make CAR T-cell therapy more efficient. In a preclinical study, investigators at UCLA generated CAR T-cells targeting TYRP1, a protein abundantly present in melanoma cells. The models demonstrated that TYRP1-specific CAR T-cells eliminate malignant cells without inducing severe adverse effects [114]. A separate phase I clinical trial is currently underway studying CAR T-cells targeting IL13Ra2, a protein expressed in melanoma in 25% of patients. The purpose is to investigate safety and efficacy in patients with advanced, treatment-refractory melanoma [115,116]. Despite the advancements in the research, the obstacles of the complex tumor microenvironment, antigen heterogeneity, and off-target therapy effects remain. Future research may investigate how to improve tumor infiltration, and how CAR T-cell therapy may offer synergistic effects when combined with other forms of immunotherapy such as PD-1/PD-L1 inhibitors, mRNA vaccines, T-VEC, and TIL therapy. 

Fundamental in considering the path ahead for these treatment modalities is addressing the unfortunate truth that access to these advanced cancer treatments is influenced by various social determinants of health that extend beyond cost. Geographic barriers, such as the limited availability of specialized cancer centers in rural or low-income urban areas, can prevent patients from receiving these potentially life-saving therapies [117,118]. Health literacy and language barriers further complicate access, as patients may struggle with the convoluted healthcare systems that often accompany advanced cancer treatments [119,120]. Finally, despite improved efforts to increase diversity, people of color still remain underrepresented and underserved in healthcare, leading to racial and ethnic disparities in cancer screening, clinical trials, and specialized care for underserved populations [121,122,123]. In pursuing groundbreaking therapeutic advancements, it is essential to ensure that no individual is excluded from their benefits due to their identity or socioeconomic status. Thus, improved healthcare infrastructure, culturally competent care, and expanded social support services are imperative in ensuring that these advancements in melanoma treatment do not leave behind certain members of the population.

## 8. Conclusions

Advancements in melanoma treatment via immunotherapies such as PD-1 inhibitors, T-VEC, mRNA vaccines, and TIL therapy mark a transformative era in oncology. While these approaches enhance immune targeting and improve prognosis, limitations like tumor resistance and logistical challenges hinder their widespread adoption. Integrative strategies leveraging the unique strengths of each modality show the potential to improve patient outcomes. Overcoming barriers to accessibility and cost-effectiveness is crucial to making these therapies universally viable. Future research should focus on optimizing treatment combinations, limiting adverse effects, and incorporating innovative manufacturing techniques to ensure equitable and effective melanoma care.

## Figures and Tables

**Table 1:** Comparison of combination immunotherapies for melanoma and their characteristics.

Combination Therapy	Efficacy	Adverse Events	Contraindications	Indications
MEK Inhibitors + PD-1/PD-L1 Inhibitors	Mixed results; some studies show improved survival, while others show no significant advantage over monotherapy.	Common AEs: rash (53.1%), diarrhea (46.9%), and fatigue (43.8%). Triple therapy increases immune-related toxicity.	Limited efficacy in some patient subsets; increased toxicity with triplet therapy.	BRAF V600-mutant metastatic melanoma; considered in high-risk cases.
CTLA-4 Inhibitors + PD-1/PD-L1 Inhibitors	Significant improvement in OS and PFS compared to monotherapy (CheckMate-067: 3-year OS 58% vs. 52% nivolumab alone).	High toxicity: grade 3–4 AEs in 59% of patients, including colitis (11%), hypophysitis (2%), and diarrhea (4%).	High-risk patients should be monitored closely for severe immune-related AEs.	Advanced melanoma, particularly in treatment-naive patients.
mRNA Vaccine + PD-1 Inhibitors	KEYNOTE-942: recurrence hazard ratio 0.561, 18-month RFS 79% vs. 62% for pembrolizumab alone.	Similar AE profile to pembrolizumab alone; 36% of patients reported AEs.	Contraindicated in patients with severe autoimmune disease due to risk of immune activation.	High-risk resected melanoma (stage II–IV); enhances pembrolizumab efficacy.
TIL Therapy + PD-1 Inhibitors	ORR 36% in combination trials, but prior anti-PD-1 exposure may reduce efficacy.	Mild toxicity but response may be lower in PD-1-experienced patients.	Patients previously exposed to anti-PD-1 therapy may have lower response rates.	Metastatic melanoma with sufficient tumor burden for TIL harvest.
TIL Therapy + mRNA Vaccines	No clinical trials reported yet; potential for enhanced immune response.	Unknown; no clinical trials have been conducted yet.	Not yet established; future trials needed.	Potential future indication for metastatic melanoma if proven effective.

## Data Availability

Not applicable.
